# *MCAM* is associated with metastasis and poor prognosis in osteosarcoma by modulating tumor cell migration

**DOI:** 10.1002/jcla.24214

**Published:** 2021-12-27

**Authors:** Xiaotian Du, Qin Zhang, Siyuan Wang, Xiao Chen, Yue Wang

**Affiliations:** ^1^ Spine lab Department of Orthopedic Surgery The First Affiliated Hospital Zhejiang University School of Medicine Hangzhou China; ^2^ Department of Pharmacy The Affiliated Hospital of Hangzhou Normal University Hangzhou China; ^3^ Key Lab of Tissue Engineering and Regenerative Medicine of Zhejiang Province Zhejiang University School of Medicine Hangzhou China

**Keywords:** cell migration, *MCAM*, osteosarcoma, prognostic biomarker, survival

## Abstract

**Background:**

Although there are standard treatment options for osteosarcoma (OS), the prognoses of patients with OS remain varied. Therefore, it is important to profile OS patients at a high risk of mortality to develop focused interventions. Although tumor biomarkers are closely associated with clinical outcomes, data on prognostic biomarkers for OS remain scarce.

**Methods:**

We collected RNA expression profiles and clinical data of 90 OS patients from the GEO database (dataset GSE21257 and GSE39055) and 96 patients in the TARGET program. The data were analyzed using univariate Kaplan‐Meier survival analysis to screen candidate gene sets that might be associated with OS survival.

**Results:**

Our analysis demonstrated that melanoma cell adhesion molecule (*MCAM*) was associated with overall survival of patients with OS in the three cohorts. The data showed that *MCAM* was upregulated in OS patients who had metastases within 5 years compared to those without metastases. GO analysis revealed that genes correlated with *MCAM* were mainly involved in cell migration and wound healing processes. In addition, wound healing assays and gene set enrichment analysis results from RNA sequencing data of small interfering (si)‐*MCAM*‐transfected OS cells demonstrated that *MCAM* modulated tumor cell migration.

**Conclusions:**

Our data demonstrate that *MCAM* may be a novel prognostic biomarker for OS. *MCAM* is associated with increased cell migration ability and risk of metastasis, thus leading to poor prognoses in OS patients.

## Graphical abstract

In this study, we utilized the GEO and TARGET osteosarcoma (OS) databases and identified melanoma cell adhesion molecule (*MCAM*), a gene with multiple biological functions in OS pathology, as a novel prognostic biomarker for OS. For the first time, the prognostic role of *MCAM* in OS was demonstrated using clinical data and RNA expression profiles of multiple cohorts. Furthermore, *MCAM* and related pathways were associated with increased cell migration ability and wound healing processes, resulting in increased risk of metastasis and poor prognosis in OS patients.

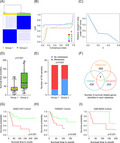

## 1 INTRODUCTION

Osteosarcoma (OS) is the most common primary bone cancer.^1^ Although the current standard treatment options for OS, such as surgical resection, chemotherapy, and radiotherapy, have substantially improved the survival rate for OS patients,^2^ a significant percentage of OS patients succumb to tumor‐related early deaths.^3^ Indeed, data have shown that OS prognosis varies considerably among patients.^4^

Obtaining the profile of OS patients at high risk of metastasis or mortality is important to help clinicians administer individualized interventions and improve clinical outcomes. Previous data have shown that assigned male sex, tumor at the axial site, large tumor size, poor response to initial treatments, elevated alkaline phosphatase levels, metastases, pathological fractures, and <90% tumor necrosis after neoadjuvant chemotherapy are associated with poor OS outcomes.^5^, ^6^, ^7^

Advanced studies have highlighted that genetic tumor biomarkers may be closely associated with clinical outcomes. For instance, in prostate tumors, a three‐gene panel of *FGFR1*, *PMP22*, and *CDKN1A* can accurately predict the risk of tumor recurrence.^8^ In OS, increased expression of *APE1* and *MDR1* was shown to be associated with poorer OS prognosis.^9^, ^10^ In addition, a risk signature of three survival‐associated genes, *MYC*, *CPE*, and *LY86*, could discriminate between low‐ and high‐mortality risk in OS patients.^11^ Unfortunately, data on prognostic biomarkers for OS remain unsatisfactory for any clinical use.

Using global gene expression profiles in multiple OS cohorts, we analyzed the clinical and RNA sequencing (RNA‐Seq) data of OS cohorts from three clinical centers. The analysis showed that melanoma cell adhesion molecule (MCAM), a transmembrane glycoprotein, could be a prognostic biomarker of OS. In addition, we evaluated the possible pathological mechanisms of *MCAM* in the prognosis of OS.

## 2 MATERIALS AND METHODS

### 2.1 Data collection

Data were downloaded from the Gene Expression Omnibus (GEO) database (including dataset GSE21257, last update date: March 22, 2012; dataset GSE39055, last update date: December 22, 2017) and obtained from the Therapeutically Applicable Research to Generate Effective Treatments (TARGET) program (Last update date: August 8, 2019) in April 2021.

The GEO (https://www.ncbi.nlm.nih.gov/geo/) is a gene expression database established by the National Center for Biotechnology Information (USA) containing array‐ and sequence‐based data^12^ The subsets of OS cohorts in the GEO database were searched using the key words ‘osteosarcoma,’ ‘RNA,’ and ‘survival.’ The GSE21257 dataset contained clinical information and gene expression data of 53 OS patients.^13^ The patient data included survival status, overall survival time, presence or absence of metastasis, and expression of 24,998 genes in biopsy samples. The GSE39055 dataset included 37 OS patients and data on survival status, overall survival time, and expression of 20,819 genes in the biopsy tissue samples.^14^ In both the GSE21257 and GSE39055 datasets, the OS tissue samples were obtained via biopsy prior to chemotherapy.

In addition, GSE16088 with 14 human OS tissue samples and 6 normal tissue samples (2 kidney samples, 2 liver samples, and 2 lymph node samples)^15^; GSE14359 with 18 human OS tissue samples (8 men and 10 women, age 31 ± 19.9) and 2 primary non‐neoplastic osteoblast cell samples^16^; and GSE52063 with 4 mesenchymal stem cell samples, 4 osteosarcoma stem cell samples, and 4 adherent osteosarcoma cell samples were also identified.^17^ These datasets included gene expression data but not clinical and survival data. Therefore, the datasets were used to profile the expression of screened candidate genes in the OS and normal samples.

The TARGET program (https://ocg.cancer.gov/programs/target) incorporates multiple tumor projects, such as those for acute lymphoblastic leukemia, acute myeloid leukemia, kidney tumors, neuroblastoma, and OS.^18^ We obtained clinical data and tissue samples in the OS project of the TARGET program from patients who were recruited in OS biopsy studies or clinical trials.^18^ The OS tissue samples used for microarray analysis were collected at the time of biopsy. Clinical data, including age, sex, survival status, overall survival time, and RNA expression profiles of OS patients (expression of 59,955 genes in OS tissues) were downloaded for analysis.

### 2.2 Screening of survival‐related candidate genes

Here, the overall survival time was defined as the time between the establishment of a clinical diagnosis of OS and death from all causes. Using Kaplan‐Meier (KM) survival analysis, univariate survival analysis was performed for each gene in the GEO GSE21257, TARGET, and GEO GSE39055 datasets. Genes with a *p*‐value of <0.01 in the KM survival analyses were selected as candidate genes. By determining overlapping significant candidate genes among the three datasets, gene sets potentially associated with the survival of OS patients were identified. The KM survival analysis was performed using the “survival” package in *R* software (version 3.6.2,). Based on the median expression of each candidate gene, we classified patients in high expression (higher than the median) or low expression (lower than the median) groups.

### 2.3 Expression, co‐expression, and functional enrichment analyses

Gene expression profiles in OS cell lines were obtained from the Cancer Cell Line Encyclopedia (CCLE) (www.broadinstitute.org/ccle). Biological networks of the top 10 genes that were correlated with the candidate genes were constructed using *STRING* (version 11.0, https://string‐db.org) and *CytoHubba* (*Cytoscape*, version 3.7.2, https://cytoscape.org/). Similarly, the top 100 co‐expression genes were extracted from the GEPIA2 database (http://gepia2.cancer‐pku.cn), which contains data on genes and their functions in various human cancers.

Cluster analysis was performed using the “ConsensusClusterPlus” package in *R* software. Gene Ontology (GO) functional enrichment analysis of the gene sets was performed using *Metascape* (http://metascape.org) while Gene Set Enrichment Analysis (GSEA) (https://www.gsea‐msigdb.org/gsea/index.jsp) for GO sets was performed using the “clusterProfiler” and “enrichplot” packages in *R* software.

### 2.4 Cell culture and transfection

The human OS cell line 143B was purchased from ZhongQiaoXinZhou Biotechnology (NO.ZQ0455). The cells were routinely cultured in complete culture medium (ZhongQiaoXinZhou, NO.ZQ‐303) at 37°C in a 5% CO_2_ atmosphere.

Transfection was performed at a cell density of 60% in a six‐well plate with small interfering (si) RNA targeting *MCAM* (si‐*MCAM*) or non‐specific control siRNA (si‐con; Santa Cruz Biotechnology; NO.sc‐35918 and sc‐37007, respectively) using Lipofectamine 2000 transfection reagent (Invitrogen; Thermo Fisher Scientific; NO.11668027) for 48 h, following the manufacturer's protocol. Thereafter, the effect of siRNA transfection was examined using western blot analysis, and the cells were used for subsequent experiments.

### 2.5 Western blot analysis

Total proteins were extracted from the cells using radio immunoprecipitation assay lysis buffer (Beyotime Institute of Biotechnology; NO.P0013). The protein concentration was estimated using a BCA protein assay kit (Pierce; No.23227). The protein samples (20 µg/lane) were resolved by 10% SDS‐PAGE and transferred onto a polyvinylidene fluoride membrane. The blots were incubated with either primary anti‐MCAM (Abcam, Cambridge; NO.ab75769) or anti‐GAPDH (Beyotime Institute of Biotechnology; NO.AG019) antibodies and diluted with Tris‐buffered saline and Tween‐20 for 2 h at 25°C. The blots were washed and then incubated with a secondary antibody (Abcam, Cambridge; NO.ab6721) for 1 h at 25°C. Western blot detection reagents (enhanced chemiluminescence; Beyotime Institute of Biotechnology; NO.P0018S) were used to generate a chemiluminescent signal according to the manufacturer's protocol.

### 2.6 Wound healing assay

The cells transfected with si‐*MCAM* or si‐con were cultured in a 6‐well plate (2.5 × 10^6^ cells/well). Using a 100 µl plastic pipette tip, each well was scratched. The cells were washed three times with PBS and cultured for 24 h. Using an inverted microscope, we captured images of the wounded area immediately after the scratch and after 24 h (magnification × 10). The gap area of each culture well was measured using the *Image*‐*Pro Plus* program (version 7.0, Media Cybernetics), and the mean values from the wells were obtained for analysis.

### 2.7 RNA‐Seq analysis

The cells were lysed using TRIzol (Invitrogen, No. 15596018) and prepared for RNA‐Seq. RNA‐Seq procedures including quality inspection, database construction, sequencing, mapping, and preliminary analysis were commissioned from BGI Company. We performed differential expression analysis using the “limma” package in *R* software with an adjusted *p*‐value of <0.05 and |log_2_FC|≥1.

### 2.8 Statistical analysis

KM univariate survival analysis was used to screen survival‐related candidate genes in OS. Quantitative data are presented as the mean ± SD or mean ± SE. Unpaired two‐tailed t tests were used for the statistical analysis.

## 3 RESULTS

### 3.1 Gene expression signature in OS biopsies revealed potential prognostic values

**FIGURE 1:**
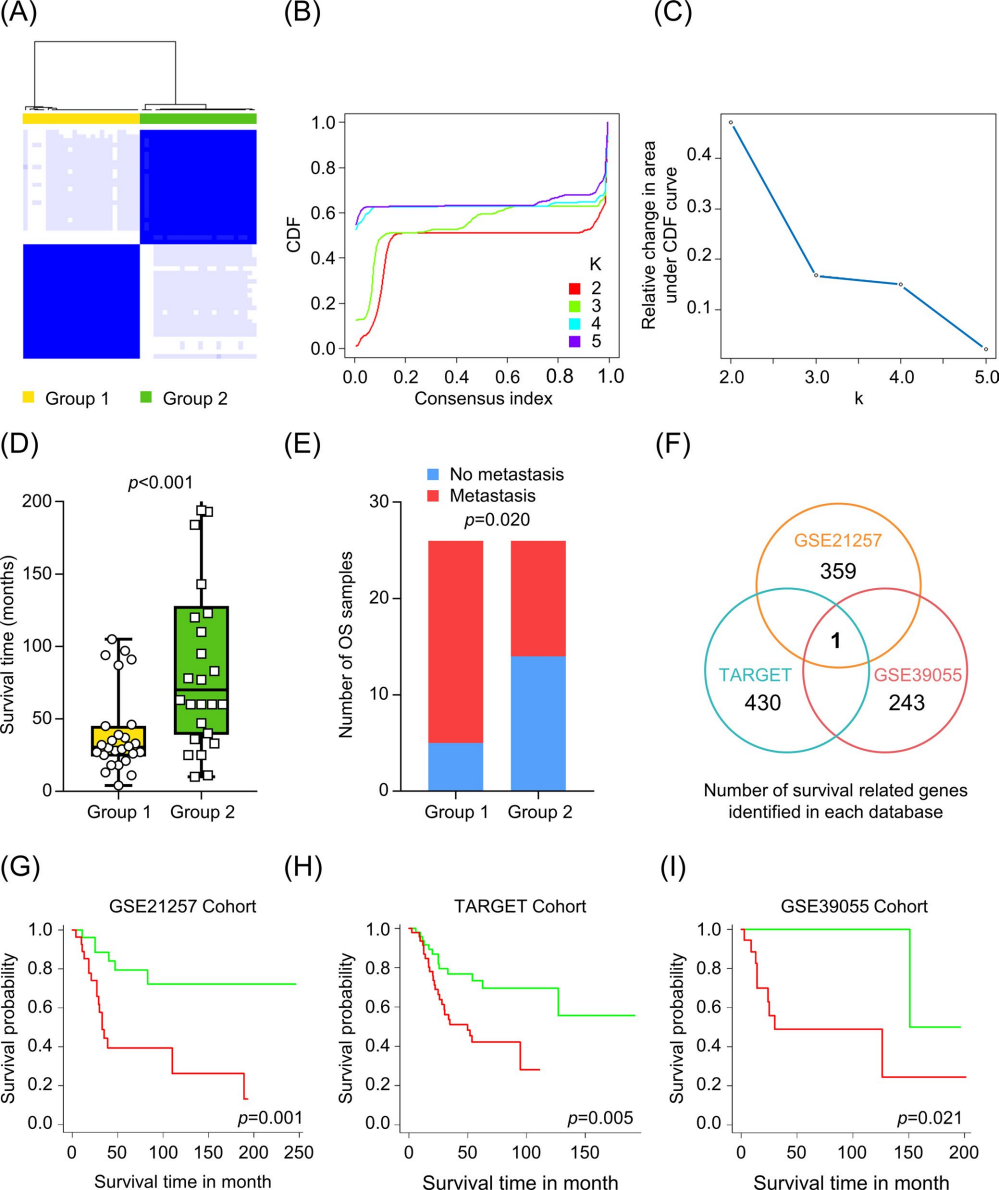
Screening for candidate genes in relation to OS prognosis. (A) Cluster analysis for biopsy samples of OS patients in GSE21257. (B) Cluster consensus values and cumulative distribution function (CDF) with different subgroup separations. (C) Relative change in area under CDF curve with different subgroup separations. (D) Patient survival time in OS subgroups. (E) Proportion of metastases at 5 years follow‐up in various OS subgroups. (F) Venn diagram of OS survival‐related genes revealed that *MCAM* was the only candidate gene that was associated with prognosis. KM survival curves for high‐ (red line) and low‐ (green line) expression of *MCAM* in the GSE21257 dataset (G), TARGET database (H), or GSE39055 dataset (I). High or low *MCAM* expression was defined according to the median expression level of the *MCAM* in each dataset

To identify possible subgroups with diverse gene expression signatures, we analyzed the gene expression profiles of 53 OS samples from GSE21257. Unsupervised clustering analysis using “ConsensusClusterPlus” revealed two distinct subgroups in GSE21257 (Group 1, *n* = 26; Group 2, *n* = 26) (Figure 1A–C). Only one sample (GSM531298) did not show concordance with either group nor was included in the subsequent analysis. Patients in Group 1 had a significantly shorter survival time (Figure 1D) and a significantly higher proportion of metastases at the 5 years follow‐up (Figure 1E) period compared to those in Group 2. These data suggest the potential prognostic value of gene expression signatures in pre‐chemotherapy biopsy samples. The survival‐related candidate genes were further explored using OS datasets in the GEO database (GSE21257, *n* = 53; GSE39055, *n* = 37) and the TARGET program (*n* = 96) (Table 1).

**TABLE 1:** Clinical characteristics of osteosarcoma (OS) patients studied in survival‐related candidate gene screening

Variable	TARGET OS cohort (*n* = 96)	GEO OS cohort	
		GSE21257 (*n* = 53)	GSE39055 (*n* = 37)
Age (years)*	15.4 ± 5.3	18.7 ± 12.2	13.5 ± 11.2
Gender			
Female	40 (41.7%)	19 (35.8%)	17 (45.9%)
Male	56 (58.3%)	34 (64.2%)	20 (54.1%)
Primary Tumor Site			
Arm/Hand	7 (7.3%)	8 (15.1%)	N/A
Leg/Foot	84 (87.5%)	44 (83.0%)	N/A
Other	5 (5.2%)	1 (1.9%)	N/A
Metastasis			
No	73 (76.0%)	39 (73.6%)	N/A
Yes	23 (24.0%)	14 (26.4%)	N/A
Survival Status			
Survival	58 (60.4%)	30 (56.6%)	27 (73.0%)
Death	38 (39.6%)	23 (43.4%)	10 (27.0%)
Overall survival time (months)*	47.6 ± 36.2	68.5 ± 59.3	52.9 ± 49.5

Note

Data are number of cases and percentages.

*Data are Mean ± SD. N/A: Not available.

### 3.2 Higher MCAM expression associated with poor OS prognosis

Among the genes that were expressed in the tissue samples from the GSE21257 OS cohort, 359 were significantly associated with overall survival time (univariate KM analysis, *p* < 0.01). Similarly, 430 and 243 survival‐related genes were identified in the TARGET and GSE39055 OS cohorts, respectively (univariate KM analysis, *p* < 0.01). Through intersection of the survival‐related genes identified in the three OS cohorts, we show that only the *MCAM* gene is associated with overall survival time (Figure 1F). Notably, higher expression of the *MCAM* gene was associated with worse prognosis of OS patients in each of the studied OS cohorts (Figure 1G–I).

### 3.3 MCAM promotes cell migration

**FIGURE 2:**
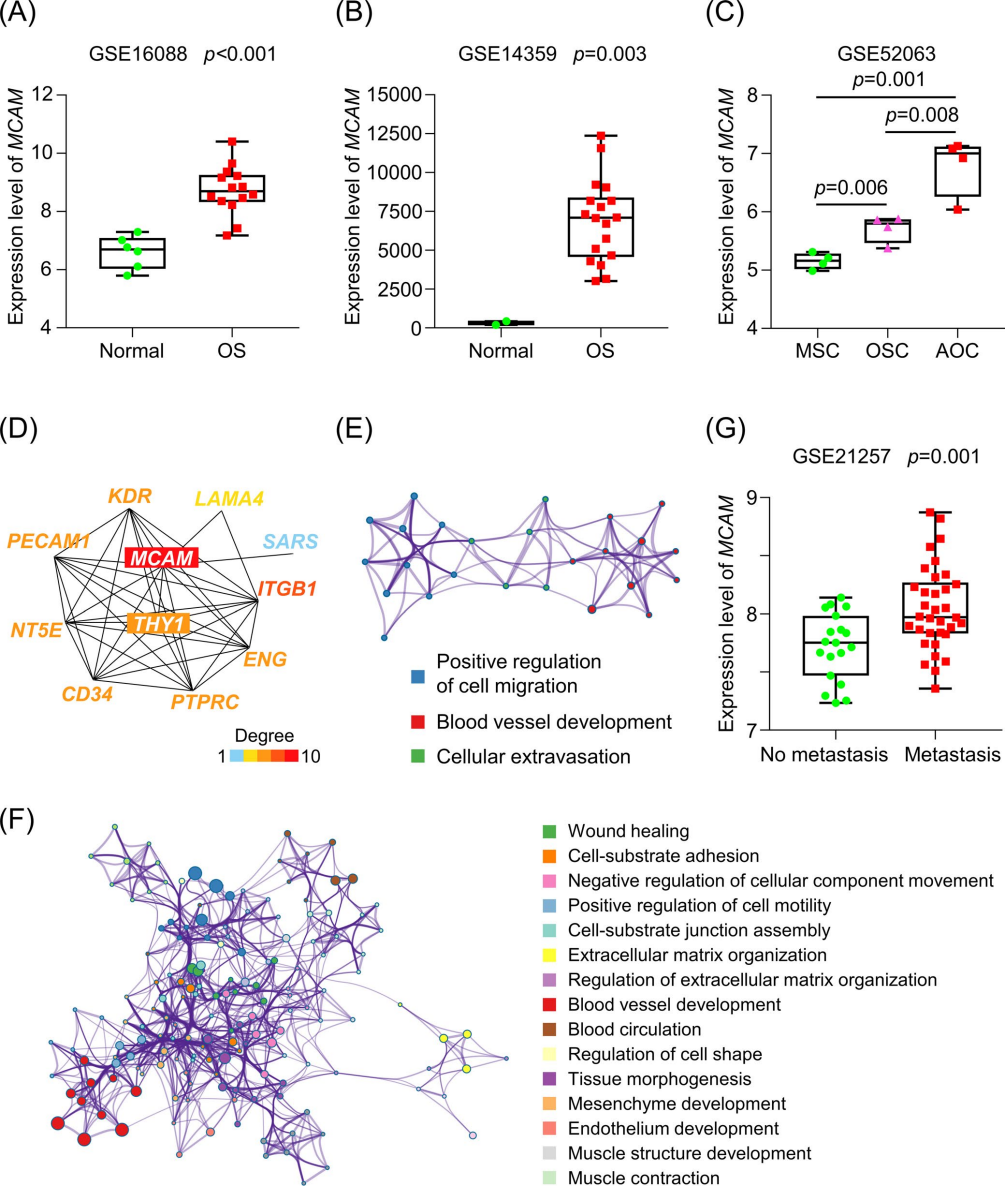
High *MCAM* expression was associated with poor OS prognosis through promoting tumor cell migration. (A) *MCAM* expression was significantly higher in human OS tissue samples compared to normal tissue samples (GSE16088, *n* = 14 and 6, respectively). (B) *MCAM* expression was significantly higher in the human OS tissues compared to primary non‐neoplastic osteoblast cells (GSE14359, *n* = 18 and 2, respectively). (C) *MCAM* expression was significantly higher in osteosarcoma stem cells (OSC, *n* = 4) and adherent osteosarcoma cells (AOC, *n* = 4) compared with mesenchymal stem cells (MSC, *n* = 4) (GSE52063). (D) Network of the top 10 genes correlated with *MCAM*. (E) GO analyses in *Metascape* for the top 10 genes that correlated with *MCAM*. (F) GO analyses in *Metascape* for the top 100 co‐expressed genes with *MCAM*. (G) *MCAM* expression in OS patients with and without metastasis (GSE21257, *n* = 34 and 19, respectively). Unpaired two‐tailed t test was used for the analysis

To understand the pathological roles of *MCAM* in OS, we assessed its expression in human OS and normal samples in the GES16088, GSE14359, and GSE52063 datasets. In GES16088, the human OS tissue (*n* = 14) showed higher *MCAM* expression compared to normal tissue samples (*n* = 6) (Figure 2A). In GSE14359, 18 human OS tissue samples showed higher expression of *MCAM* than that in two primary non‐neoplastic osteoblast cell samples (Figure 2B). In addition, *MCAM* expression was successively increased in mesenchymal stem cell samples (MSC, *n* = 4), osteosarcoma stem cell samples (OSC, *n* = 4), and adherent osteosarcoma cell samples (AOC, *n* = 4) (Figure 2C). Taken together, these data demonstrated substantial upregulation of *MCAM* expression in human OS samples compared to the normal samples.

The top 10 *MCAM* correlated genes and the top 100 *MCAM* co‐expression genes were then extracted from the *STRING* and GEPIA2 databases, respectively. The GO analyses with *Metascape* revealed that the top 10 genes correlated with *MCAM* were mainly associated with positive regulation of cell migration (Figure 2D,E). The top 100 *MCAM* co‐expression genes were shown to be involved in wound healing, positive regulation of cell motility, and cell‐substrate adhesion in *Metascape* GO analyses (Figure 2F). Among the 53 OS patients in the GEO GSE21257 dataset, 34 (64.2%) had metastases at the 5 years follow‐up, while 19 (35.8%) did not. Notably, *MCAM* expression in the biopsies was significantly higher in patients who had metastases within 5 years than in those without metastases (Figure 2G).

### 3.4 MCAM knockdown impaired OS cell migration

**FIGURE 3:**
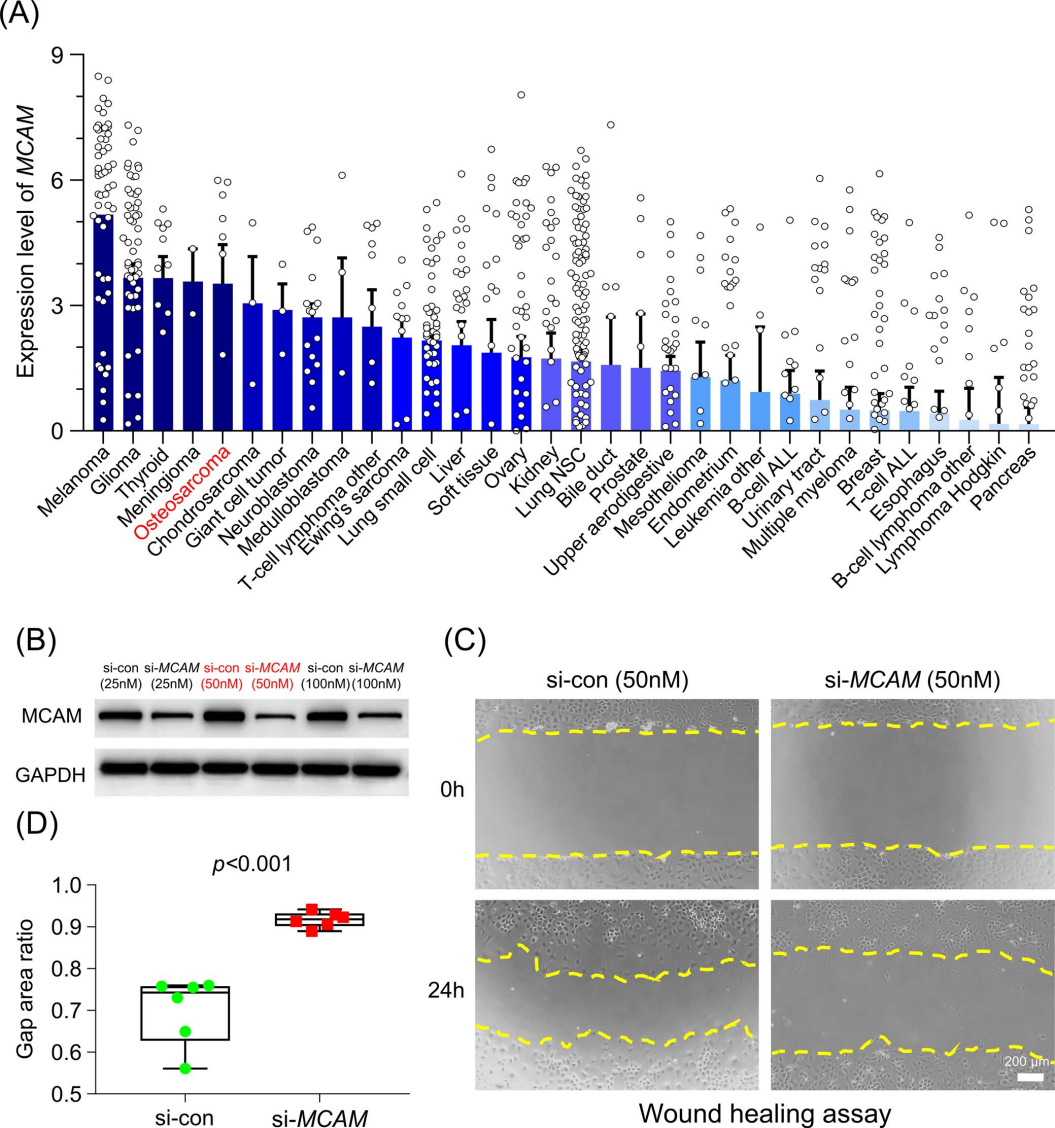
*MCAM* knockdown impaired the migration ability of OS cells. (A) *MCAM* was highly expressed in OS cell lines according to CCLE. (B) The transfection efficiency of different concentrations of si‐*MCAM* in 143B cells. (C) Scratch wounds of 143B cells at 0 and 24 h after transfecting with si‐*MCAM* or si‐con. (D) Average gap area ratios of scratch wounds at 0 and 24 h after transfection. Unpaired t test was used for the analysis. CCLE: Broad Institute Cancer Cell Line Encyclopedia. si‐*MCAM*, small interfering RNA targeting *MCAM*; si‐con, small interfering RNA of non‐specific control

The expression levels of *MCAM* in OS cell lines were determined using the Broad Institute CCLE (Figure 3A). siRNA was used to knockdown *MCAM* in the human OS cell line 143B. A concentration of 50 nM *MCAM* siRNA, with a relatively high knockdown efficiency, was used in experiments to evaluate the migration ability and facilitate gene expression analysis in OS cells (Figure 3B). A wound healing assay was performed to assess the migration ability of cells *in vitro*. As presented in Figure 3C and 3D, *MCAM* knockdown significantly impaired OS cell migration.

### 3.5 MCAM modulates multiple biological processes in OS

**FIGURE 4:**
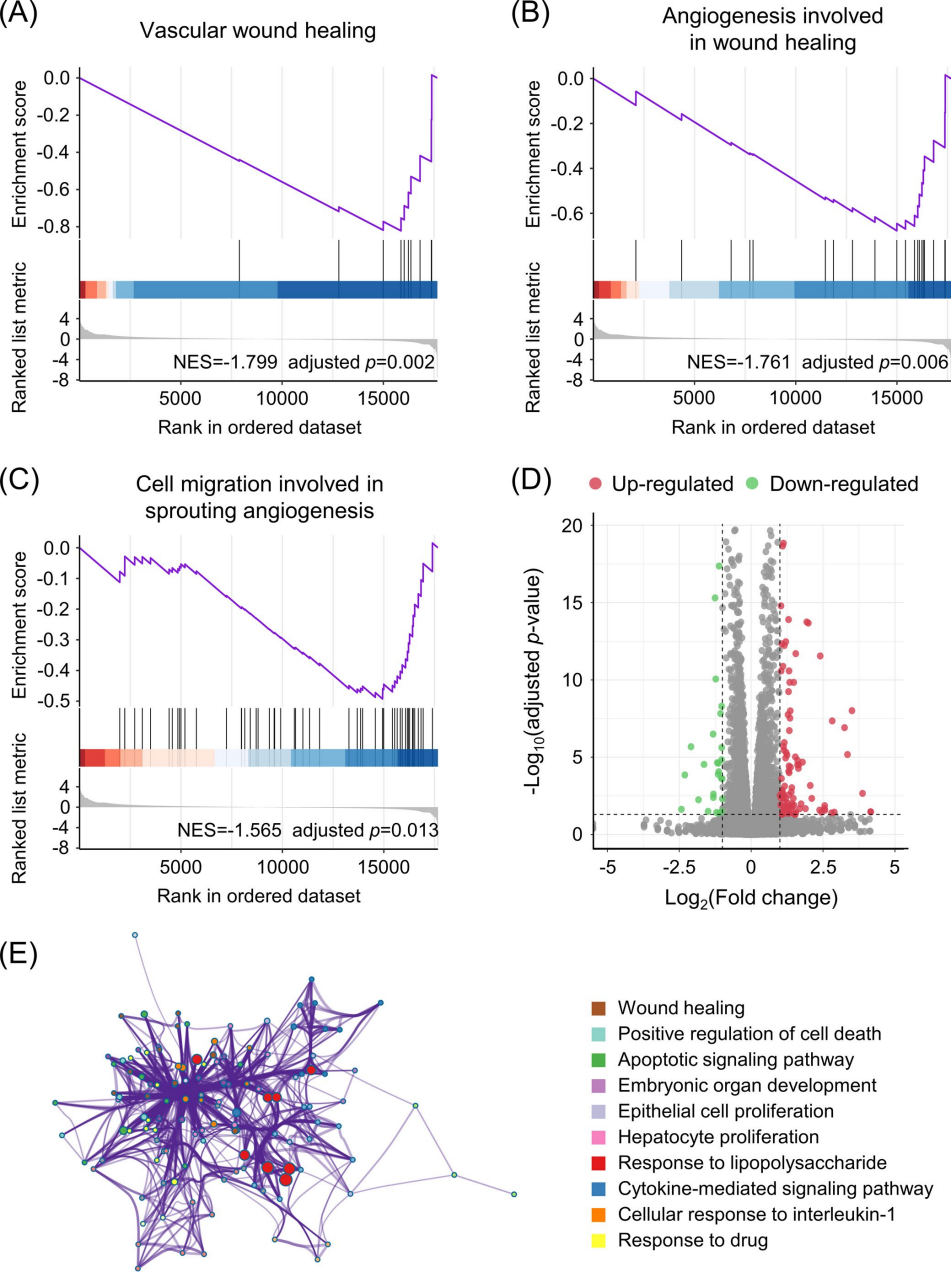
*MCAM* modulates various biological processes in OS. Gene Set Enrichment Analysis (GSEA) of gene sets for vascular wound healing (A), angiogenesis involved in wound healing (B), and cell migration involved in sprouting angiogenesis (C). (D) Up‐ and downregulated genes in si‐*MCAM* 143B cells. (E) Downregulated GO terms in *Metascape* of the 143B cells for si‐*MCAM* cell

To understand the possible mechanisms of *MCAM* in OS prognosis, we used RNA‐Seq analysis to study the gene expression profiles in si‐*MCAM* and si‐con transfected cells. GSEA revealed that gene sets involved in vascular wound healing, angiogenesis involved in wound healing, and cell migration involved in sprouting angiogenesis were significantly downregulated in si‐*MCAM* cells (Figure 4A–C).

In addition, in the si‐*MCAM* cells, 106 and 43 genes were significantly up‐ and downregulated, respectively (|log_2_FC|≥1, adjusted *p*‐value <0.05, Figure 4D). GO analysis with *Metascape* of the differentially expressed genes revealed that genes responsible for wound healing, positive cell death regulation, apoptotic signaling pathway function, cell proliferation, and cytokine‐mediated signaling pathway function were significantly downregulated in si‐*MCAM* cells, whereas no GO terms were enriched for upregulated genes (Figure 4E).

### 3.6 Roles of MCAM in other cancers

The possible roles of *MCAM* in other cancers were further investigated using the GEPIA2 database. The data showed upregulation of the *MCAM* gene in samples from cholangiocarcinoma, lymphoid neoplasm diffuse large B‐cell lymphoma, glioblastoma multiforme, head and neck squamous cell carcinoma, kidney renal clear cell carcinoma, liver hepatocellular carcinoma, pancreatic adenocarcinoma, skin cutaneous melanoma, and thymoma, compared with adjacent normal tissue samples (|log_2_FC|≥1, adjusted *p*‐value <0.05, for all tumors; Figure S1).

In addition, survival analyses demonstrated that higher *MCAM* expression was significantly associated with worse prognosis in patients with brain low‐grade glioma (HR = 2.2, adjusted *p*‐value <0.05) and mesothelioma (HR = 2.8, adjusted *p*‐value <0.05) compared to other patients.

## 4 DISCUSSION

Although there are standard treatment options for OS, there is variance in responsiveness and prognosis in OS patients. One possible approach to improve the clinical outcomes of OS patients is to define reliable prognostic biomarkers of OS that could precisely identify patients at high risk of mortality for focused clinical interventions. Here, we utilized the GEO and TARGET OS databases and identified *MCAM*, a gene with multiple biological functions in OS pathology, as a novel prognostic biomarker for OS. The expression of *MCAM* and related pathways was associated with increased cell migration ability and wound healing processes, which were correlated with an increased risk of metastasis and poor prognosis in OS patients.

*MCAM*, also known as MUC18, Mel‐CAM, CD146, A32 antigen, or S‐Endo‐1, is a transmembrane glycoprotein that acts as a Ca^2+^‐independent adhesion molecule^19^ and can mediate cellular adhesion by regulating cell‐cell and cell‐matrix interactions.^20^ High expression of MCAM is a common phenomenon in a number of normal human tissues, including hair follicles,^21^ retina,^22^ endotheliocyte,^23^ and mammary ducts.^24^ Although the functions of *MCAM* have yet to be fully illustrated, *MCAM* has been shown to play an important role in cell migration. For example, *MCAM* promotes the migration of lymphocytes to secondary lymphoid organs^24^ and enhances migration and differentiation processes in neural stem cells.^25^ In addition, *MCAM* promotes the development of pigment epithelium by promoting the migration of retinal cells.^26^

In addition, high *MCAM* expression has also been demonstrated in tumor tissues, such as melanoma,^27^ hepatocellular carcinoma,^28^ gastric carcinoma,^29^ and breast cancer^30^ and thus was thought to mediate tumor development and prognosis. For instance, *MCAM* overexpression promotes tumor cell migration, tumor invasion, and cancer stem cell‐like activities in triple‐negative breast cancer.^30^ Moreover, *MCAM* has been associated with poor clinical outcome due to enhanced development and progression of tumor tissues in gastric malignancies.^29^ Considering this evidence, *MCAM* has been suggested as a potential immunotherapeutic target against *MCAM*‐positive tumors. In fact, antibody‐ and vaccine‐based strategies targeting *MCAM* have been proposed for treating melanoma^31^ as well as for ovarian, cervical, and liver cancers.^32^, ^33^

Similarly, *MCAM* has been shown to be highly expressed in various OS cell lines, such as SaOS, MG‐63, and U‐2OS^34^ as well as in the circulating endothelial cells of OS tissue samples.^35^ Echoing previous findings, our study demonstrated that *MCAM* was highly expressed in both human OS tissues and 143B OS cells. For the first time, this study collected clinical data and RNA expression profiles from multiple OS cohorts and revealed that *MCAM* expression was associated with poor OS prognosis. Our findings demonstrate that *MCAM* may be an effective biomarker for predicting OS prognosis.

In addition, *MCAM* may be associated with metastasis in OS, which worsens the clinical prognosis. In a recent study, a considerable increase in *MCAM*‐positive macrophage cells in OS tumors with remote metastasis was observed compared to those without metastasis.^36^ In agreement, our findings showed higher expression of *MCAM* in tumor samples from OS patients who had metastases within 5 years compared to those without metastasis. More importantly, GO analysis revealed that the top 10 and top 100 genes correlated with *MCAM* were mainly involved in cell migration and wound healing processes. In addition, wound healing tests and GSEA results from the RNA‐Seq data of si‐MCAM‐transfected OS cells confirmed that the *MCAM* gene mediated the wound healing process in tumor cells. The evidence suggested that the *MCAM* gene may facilitate tumor cell migration and was associated with metastasis and poor prognosis in OS patients.

Several studies have investigated the use of anti‐*MCAM* therapies for OS. These therapies mainly targeted biological processes involving remote metastasis. ABX‐MA1, a human anti‐MCAM antibody, can inhibit spontaneous pulmonary metastasis of OS cells in mice.^37^ Another radiolabeled anti‐MCAM antibody was used to target circulating and metastatic tumor cells in an OS mice model.^38^ In addition to metastasis, the current RNA‐Seq data on si‐*MCAM*‐transfected OS cells suggested that the *MCAM* protein may also participate in the cytokine‐mediated signaling pathway and apoptosis in OS. These findings suggest that anti‐MCAM should be researched further for immunotherapy against OS.

The current study used the established GEO and TARGET databases and combined clinical data and gene expression data to screen for prognostic biomarkers for OS. Although we used data from multiple centers, the overall sample size was relatively small. Moreover, despite the use of new techniques such as RNA‐Seq, which provide huge datasets, the derived results may relate to the analysis protocol and vary among institutes. Nevertheless, the role of *MCAM* in OS pathogenesis and prognosis requires further studies using OS cohorts with large sample sizes.

## 5 CONCLUSION

Our findings demonstrate that *MCAM*, with multiple biological roles in OS pathogenesis, is a novel prognostic biomarker for OS patients. *MCAM* was associated with increased cell migration ability and a greater risk of metastasis and thus could lead to relatively poor prognoses in OS patients.

## CONFLICTS OF INTEREST

All authors declare that they have no conflict of interest.

## Supporting information

Supplementary Material

## Data Availability

The data that support the findings of this study are available from the corresponding author upon reasonable request.
