# Supplementary material for: MCAM is associated with metastasis and poor prognosis in osteosarcoma by modulating tumor cell migration
Source: J Clin Lab Anal. 2021 Dec 27;36(2):e24214. doi: 10.1002/jcla.24214 (PMC8841137; doi:10.1002/jcla.24214)
Supplement: Supplementary file 1 — Supplementary Material [file JCLA-36-e24214-s001.docx]

**
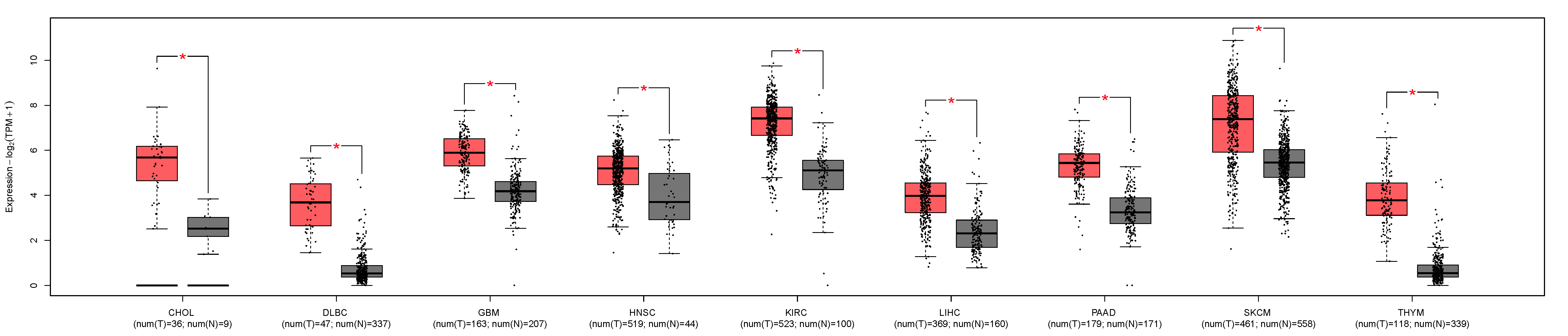
**

**Supplementary Figure 1** Various tumor samples revealed higher expression of *MCAM* than adjacent normal tissue samples in the GEPIA2 database. CHOL=Cholangio carcinoma; DLBC=Lymphoid neoplasm diffuse large B-cell lymphoma; GBM=Glioblastoma multiforme; HNSC=Head and Neck squamous cell carcinoma; KIRC=Kidney renal clear cell carcinoma; LIHC=Liver hepatocellular carcinoma; PAAD=Pancreatic adenocarcinoma; SKCM=Skin cutaneous melanoma; THYM=Thymoma.
